# Clinical outcomes of injectable and conventional pulmonary valve replacement in severe pulmonary regurgitation

**DOI:** 10.55730/1300-0144.6211

**Published:** 2026-02-25

**Authors:** Sercan TAK, Murat KOÇ, Ali KUTSAL, Vehbi DOĞAN

**Affiliations:** 1 Department of Cardiovascular Surgery, Faculty of Medicine, Gazi University, Ankara, Turkiye; 2 Department of Cardiovascular Surgery, Etlik City Hospital, University of Health Sciences, Ankara, Turkiye; 3 Department of Cardiovascular Surgery, Dr. Sami Ulus Maternity and Children’s Health and Diseases Training and Research Hospital, University of Health Sciences, Ankara, Turkiye; 4 Division of Pediatric Cardiology, Department of Pediatrics, Etlik City Hospital, University of Health Sciences, Ankara, Turkiye

**Keywords:** Tetralogy of Fallot, pulmonary regurgitation, pulmonary valve, congenital heart defects, heart surgical procedure

## Abstract

**Background/aim:**

Pulmonary regurgitation following tetralogy of Fallot repair is a common long-term complication requiring pulmonary valve replacement. Injectable bioprosthetic valves offer a less invasive alternative to conventional surgical replacement, but comparative data in pediatric populations remain limited. This study aimed to evaluate short-term and long-term clinical outcomes of injectable pulmonary valve replacement and conventional pulmonary valve replacement in pediatric patients with severe pulmonary regurgitation following tetralogy of Fallot repair.

**Materials and methods:**

This retrospective study included 22 pediatric patients who underwent pulmonary valve replacement. Patients were divided into injectable (n = 9) and conventional (n = 13) groups based on anatomical criteria. Primary outcomes included early postoperative parameters and long-term valve function. The mean follow-up duration was 10.5 ± 2.5 years.

**Results:**

Favorable early outcomes were noted in the injectable pulmonary valve replacement cohort, including shorter intensive care unit stay (16.8 ± 6.2 vs. 37.0 ± 23.4 h, p = 0.021), reduced mechanical ventilation duration (5.2 ± 3.9 vs. 15.4 ± 11.4 h, p = 0.019), decreased chest tube drainage (206.7 ± 108.2 vs. 513.1 ± 274.1 mL, p = 0.005), and shorter hospital stay (5.4 ± 2.4 vs. 8.4 ± 3.1 days, p = 0.026). Long-term outcomes indicated similar valve function and right ventricular remodeling in the two groups, with excellent freedom from reintervention (100%) during follow-up.

**Conclusion:**

Injectable pulmonary valve replacement appears to be a safe alternative, offering favorable early outcomes and long-term valve performance comparable to conventional methods. However, clinical experience and precise patient selection remain critical for achieving optimal results.

## 1. Introduction

The dramatic evolution of tetralogy of Fallot (TOF) management has shifted the clinical focus toward optimizing long-term quality of life and managing late complications in the growing population of adult survivors [1,2]. Among late complications following TOF repair, pulmonary regurgitation (PR) is the most frequently encountered and clinically significant sequela [3]. The development of significant PR is multifactorial, with transannular patch use during initial repair being the primary contributing factor [4]. Chronic volume overload from persistent PR leads to progressive right ventricular enlargement, decreased exercise capacity, increased arrhythmia susceptibility, and elevated risk of sudden cardiac death [5,6].

While pulmonary valve replacement (PVR) is a universally accepted treatment approach for symptomatic patients with right ventricular dysfunction [7], the timing for asymptomatic patients remains debated, with increasing evidence favoring early intervention to ensure ventricular recovery [4,8].

Historically, surgical PVR via median sternotomy with cardiopulmonary bypass (CPB) constituted the only therapeutic option. While effective, this approach entails well-documented risks including bleeding, reperfusion injury, and systemic inflammatory responses [9]. Transcatheter PVR (TPVR) has emerged as an alternative offering reduced early morbidity, but its anatomical limitations include outflow tract size restrictions and vascular access requirements in small children [10,11].

In 2004, injectable bioprosthetic valves were introduced, offering surgical implantation without CPB [12]. This technology combines surgical precision with the benefits of CPB avoidance, featuring self-expanding nitinol stents with specially treated pericardium [13]. The technique allows minimal cardiac dissection while maintaining satisfactory outcomes, with documented benefits including reduced operative duration, decreased bleeding, and shorter recovery periods [12].

This study aimed to assess the short-term and long-term clinical outcomes of injectable PVR (IPVR) and conventional surgical PVR (CPVR) in pediatric patients with severe PR following previous TOF repair at our institution.

## 2. Materials and methods

### 2.1. Study design and patients

This retrospective comparative study was conducted at a single tertiary pediatric cardiac surgery center following ethics committee approval. We reviewed all pediatric patients undergoing PVR for severe PR following TOF repair with a transannular patch between January 2011 and December 2020. All procedures were performed by a single senior surgeon, with two other surgeons assisting throughout the study period.

Inclusion criteria included severe PR as determined by comprehensive echocardiography, right ventricular dilatation with right ventricular end-diastolic volume index (RVEDVI) of ≥140 mL/m^2^, and either symptomatic presentation or objective evidence of declining function in asymptomatic patients. Symptomatic patients reported exercise intolerance, dyspnea, chest pain, palpitations, and/or syncope.

IPVR was considered for patients with pulmonary annulus diameters of 15–30 mm as determined by echocardiography, as the commercially available injectable valves are produced within this specific size range. Exclusion criteria for IPVR included residual intracardiac defects requiring repair, significant tricuspid regurgitation, right ventricular outflow tract stenosis with gradient of >40 mmHg, and peripheral pulmonary artery stenosis requiring intervention.

Patients not meeting the IPVR criteria or having coexisting abnormalities underwent conventional bioprosthetic valve replacement. Final surgical approach decisions were made by multidisciplinary team consensus, with an institutional preference for IPVR for all patients whose pulmonary annulus diameters fell within the specified range and who required no concomitant cardiac procedures.

### 2.2. Surgical techniques

**Figure 1:**
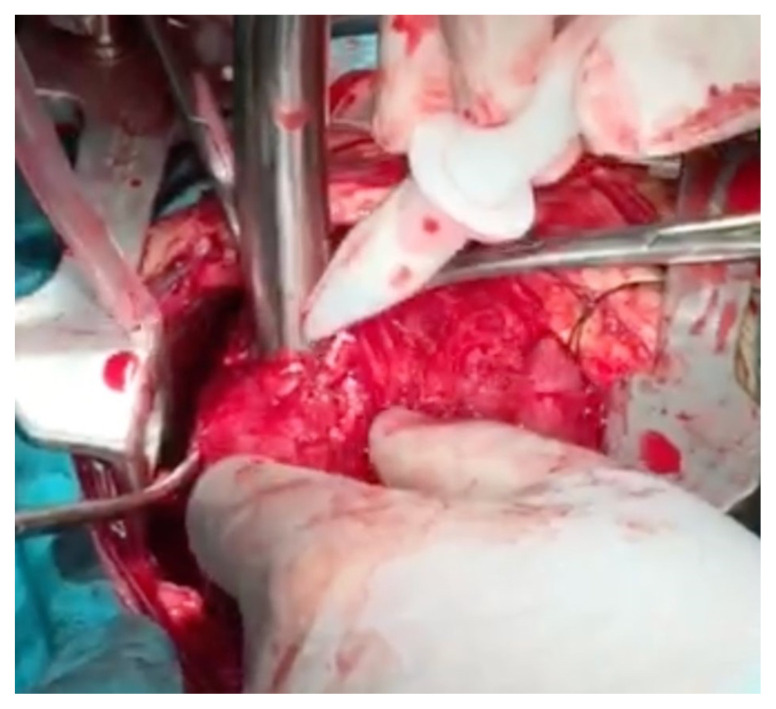
Dilation of the right ventricular outflow tract using a Hegar dilator to facilitate the advancement of the injectable valve.

**Figure 2:**
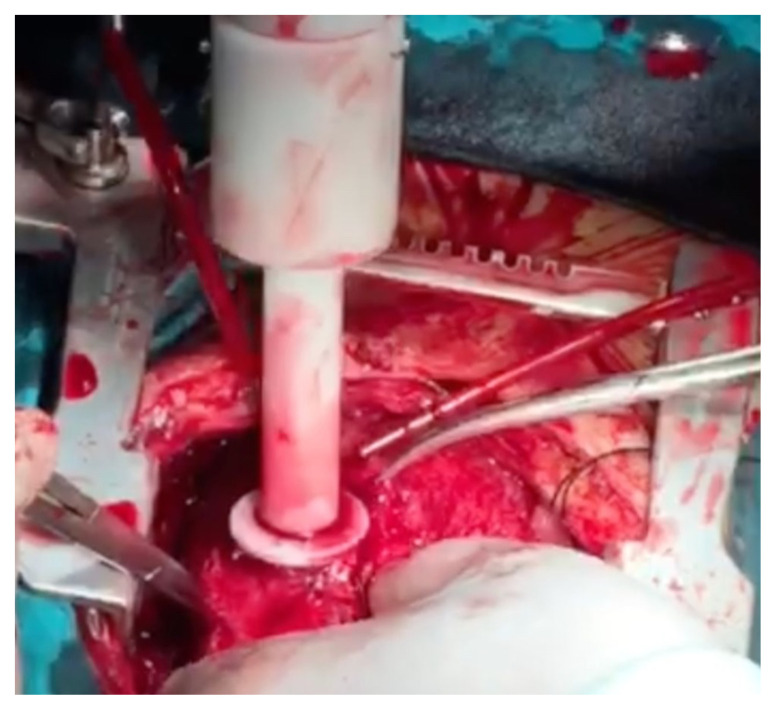
Injectable valve system placed within the right ventricular outflow tract.

For the IPVR group, the procedure was performed via sternotomy and without CPB. A limited ventriculotomy was created on the anterior surface of the right ventricle, carefully positioned away from the coronary arteries, after placing two polymer pledget-reinforced purse-string sutures. The ventriculotomy was progressively dilated with Hegar dilators to accommodate a trocar system (Figure 1). This system was then advanced through the right ventricle to the pulmonary annulus. The No-React Injectable Bioprosthetic Pulmonary Valve (Shelhigh Inc., Union, NJ, USA), sized 2 mm larger than the echocardiographically measured annulus diameter, was advanced over the trocar and precisely positioned within the pulmonary outlet (Figure 2). After ensuring that the pulmonary valve’s stent did not extend into the right or left pulmonary artery ostia, the trocar was removed. The ventriculotomy was then closed using the preplaced purse-string sutures. Intraoperative transesophageal echocardiography was utilized to assess valve function, the right ventricular outflow tract, and pulmonary artery branches. Following confirmation of normal valve function and position, the valve stent was secured to the pulmonary artery with two Prolene fixation sutures to prevent migration. Hemostasis was subsequently verified, concluding the procedure.

CPVR procedures entailed CPB with mild hypothermia. Following any concomitant repairs, St. Jude Medical Trifecta bioprosthetic valves (Abbott, Abbott Park, IL, USA) were implanted using standard techniques. Isolated valve replacement procedures were performed with a beating-heart technique and CPB support. However, in the presence of concomitant cardiac defects requiring the opening of heart chambers, procedures were performed under aortic cross-clamping.

Comprehensive echocardiographic evaluations were performed intraoperatively, on postoperative day 1, at discharge, 6 months postoperatively, and annually thereafter. Primary outcome measures included early postoperative parameters and long-term valve function.

### 2.3. Definitions of perioperative variables

The vasoactive inotropic score (VIS) was assessed using the VIS 2020 formula proposed by Belletti et al. [14]: VIS = dopamine (μg/kg/min) × 1 + dobutamine (μg/kg/min) × 1 + epinephrine (μg/kg/min) × 100 + norepinephrine (μg/kg/min) × 100 + vasopressin (U/kg/min) × 10,000 + milrinone (μg/kg/min) × 10 + enoximone (μg/kg/min) × 1 + levosimendan (μg/kg/min) × 50 + olprinone (μg/kg/min) × 25 + methylene blue (mg/kg/h) × 20 + phenylephrine (μg/kg/min) × 10 + terlipressin (μg/kg/min) × 10 + angiotensin II (ng/kg/min) × 0.25.

Chest tubes were removed when the drainage amount had decreased below 2 mL/kg over the preceding 12 h, provided there were no additional pulmonary issues requiring drainage. The total drainage volume was calculated as the cumulative amount collected until the time of tube removal.

### 2.4. Statistical analysis

Statistical analyses were performed using IBM SPSS Statistics 23.0 for Windows (IBM Corp., Armonk, NY, USA). Continuous variables were expressed as mean ± standard deviation for normally distributed data or median (interquartile range [IQR]) for nonnormally distributed data. Categorical variables were presented as frequencies and percentages.

The Shapiro–Wilk test was used to assess the normality of continuous variables. For normally distributed continuous variables, the two-sample Student t-test was employed to compare means between groups. For nonnormally distributed data, the Mann–Whitney U test was utilized. Categorical variables were compared using the chi-square test or Fisher exact test as appropriate based on expected cell frequencies.

All statistical tests were two-tailed and values of p < 0.05 were considered statistically significant. Given the exploratory nature of this study and the limited sample size, no adjustment for multiple comparisons was applied and results should be interpreted with appropriate caution.

## 3. Results

### 3.1. Patient demographics and baseline characteristics

Between 2011 and 2020, 22 pediatric patients underwent PVR; 9 (40.9%) received IPVR and 13 (59.1%) received CPVR. The cohort included 13 boys (59.1%) and 9 girls (40.9%). The sex distribution was similar between the groups (p = 1.000). Mean age at PVR was 10.8 ± 2.7 years, with no significant difference between the IPVR (10.3 ± 3.2 years) and CPVR (11.2 ± 2.4 years) groups (p = 0.503). Similarly, the time interval between the initial corrective surgery and subsequent PVR was comparable between the two groups [median (IQR): 8.0 (2.0) years vs. 9.0 (5.0) years for IPVR and CPVR, p = 0.591). Detailed baseline characteristics are presented in Table 1.

### 3.2. Preoperative assessment

Eighteen patients (81.8%) were symptomatic at presentation, with exercise intolerance being most common (68.2%), followed by dyspnea (54.5%). The preoperative RVEDVI was 142.3 ± 30.2 mL/m^2^ in the IPVR group and 157.5 ± 23.0 mL/m^2^ in the CPVR group (p = 0.195). Patients treated with IPVR had significantly lower right ventricular end-systolic volume index (RVESVI) (90.0 ± 16.6 vs. 111.1 ± 20.6 mL/m^2^, p = 0.019), suggesting better preserved systolic function.

### 3.3. Operative characteristics

All IPVR procedures were performed without CPB, while all CPVR procedures required CPB (mean CPB time: 77.0 ± 17.4, p < 0.001). The mean implanted valve size was 26.1 ± 3.8 mm in the IPVR group and 23.6 ± 2.8 mm in the CPVR group (p = 0.089). Concomitant procedures were performed exclusively in the CPVR group, including ventricular septal defect closure (n = 3), peripheral pulmonary artery stenosis repair (n = 5), and tricuspid annuloplasty (n = 2). Detailed operative characteristics are shown in Table 2.

### 3.4. Early postoperative outcomes

One patient in the IPVR group experienced hemodynamic instability during the early postoperative period. Echocardiographic evaluation revealed displacement of the pulmonary valve toward the left pulmonary artery. This necessitated emergency surgery, wherein PVR was performed via an open surgical approach. Furthermore, the IPVR group had better early postoperative outcomes. The ICU stay was markedly shorter in the IPVR group (16.8 ± 6.2 vs. 37.0 ± 23.4 h, p = 0.021), constituting a 55% reduction. Mechanical ventilation duration was also significantly reduced (5.2 ± 3.9 vs. 15.4 ± 11.4 h, p = 0.019), with 88.9% of IPVR patients extubated within 6 h versus 38.5% of CPVR patients (Table 3). Postoperative bleeding was significantly decreased in the IPVR group (206.7 ± 108.2 vs. 513.1 ± 274.1 mL, p = 0.005), constituting a 60% reduction. Hospital stay was shorter for the IPVR group (5.4 ± 2.4 vs. 8.4 ± 3.1 days, p = 0.026), entailing a 36% reduction (Table 3).

### 3.5. Complications

No significant complications occurred in the IPVR group during this series. The CPVR group experienced a higher overall complication rate (38.5% vs. 0.0%), including prolonged ventilation, renal dysfunction, wound infection, and heart block. No early mortality occurred in either group.

### 3.6. Long-term outcomes

The mean follow-up duration was 10.3 ± 3.0 years in the IPVR group and 10.7 ± 2.3 years in the CPVR group (p = 0.751). Both techniques achieved significant right ventricular reverse remodeling. RVEDVI decreased from 142.3 ± 30.2 to 94.1 ± 17.8 mL/m^2^ in the IPVR group and from 157.5 ± 23.0 to 103.2 ± 19.9 mL/m^2^ in the CPVR group, with similar degrees of reduction between the groups (p = 0.288). During follow-up, 88.9% of IPVR patients and 76.9% of CPVR patients achieved RVEDVI of <120 mL/m^2^. Mean ejection fraction was 65.1 ± 3.7% in the IPVR group versus 61.3 ± 5.5% in the CPVR group (p = 0.087). Transvalvular gradients remained low, being 25.6 ± 9.2 mmHg in the IPVR group versus 30.9 ± 16.2 mmHg in the CPVR group (p = 0.381).

Functional capacity was excellent; 88.9% of IPVR and 84.6% of CPVR patients achieved New York Heart Association Class I results (p = 0.743). No clinically significant arrhythmias occurred during follow-up. Both groups demonstrated 100% freedom from valve-related reintervention. An isolated complication was noted in one patient from the CPVR group, who experienced a sinus of Valsalva rupture 3 years after surgery; this was successfully treated with transcatheter occlusion. No infective complications, including prosthetic valve endocarditis or deep wound infections, were observed in either group during the entire follow-up period. Comprehensive long-term results are detailed in Table 4.

## 4. Discussion

Injectable valve technology occupies a unique position between conventional surgery and transcatheter approaches, and due to its minimally invasive nature, TPVR is now applied as the first-line treatment option in selected cases. While transcatheter techniques offer complete avoidance of surgical incisions and comparable results to surgery in terms of mortality and long-term valve function [15], they have significant anatomical limitations. They are applicable in both native right ventricular outflow tract (RVOT) and conduit settings, but they are subject to size-related restrictions. Specifically, a wide RVOT is often unsuitable, particularly for balloon-expandable valves [15]. Furthermore, vascular access issues can arise in small children. The risk of coronary compression was reported more frequently in earlier studies but has significantly decreased in recent publications due to increased experience and more meticulous preprocedural assessments [16]. Another complication that may be observed following TPVR is endocarditis. Reported outcomes regarding this complication are highly variable [17–19]. For example, a review of nine studies conducted by Abdelghani et al. [17] reported endocarditis rates ranging from 3.2% to 25%. In contrast, the Compassion study [18] reported a 3-year endocarditis-free survival rate of 97.1%. Notably, we observed no cases of endocarditis in our series.

Injectable valves provide many benefits, avoiding CPB while maintaining surgical precision and versatility. The introduction of injectable bioprosthetic pulmonary valves in the early 2000s presented a significant alternative for carefully selected patients by minimizing the risks associated with CPB. These bioprostheses are engineered with a low-profile design and a flexible stent structure, which mitigates annular stretching and reduces the risk of coronary artery compression. The absence of a suture ring facilitates the implantation of relatively larger prostheses, thereby preventing RVOT obstruction and promoting laminar flow across the valve. Moreover, the implantation technique requires only minimal mobilization of the heart and great vessels, leading to shorter operative times and decreased risks associated with extensive dissection. For IPVR patients, we selected prosthetic valves that were 2 mm larger than the annular diameter measured by echocardiography. This approach was adopted to minimize the risk of paravalvular leakage and valve migration. Additionally, the use of an oversized valve may help to reduce the pressure gradient between the right ventricle and pulmonary artery, thereby mitigating long-term structural valve deterioration [20].

This study demonstrated significantly shorter ICU and hospital stays, reduced postoperative bleeding, and shorter mechanical ventilation times in the IPVR cohort. These favorable early results were expected, as avoiding CPB preserves the coagulation mechanism, reduces the systemic inflammatory response, maintains normal cardiac rhythm, and eliminates aortic complications related to cannulation [9]. Additionally, the injectable technique minimizes surgical trauma in redo cases by reducing the need for extensive cardiac dissection. However, these advantages should be interpreted with caution; they are likely attributable to the absence of CPB and the lower surgical complexity in the IPVR group rather than the inherent superiority of the valve technology itself. In contrast, the majority of patients in the CPVR group required concomitant cardiac procedures and presented with more complex intracardiac anomalies, necessitating longer operative times and a more invasive approach. It must also be acknowledged that the significantly lower preoperative RVEDVI and better-preserved right ventricular function in the IPVR cohort likely contributed to the improved early postoperative outcomes, representing a more favorable baseline status compared to the CPVR group.

Similar to our study, the INVITE trial [21], which was the first major study to compare these two techniques, evaluated the outcomes of 19 patients, with 8 in a standard PVR group and 11 in an IPVR group. According to the early outcomes of the study, no significant difference was found between the two groups, except for significantly lower 24-h chest tube drainage in favor of the IPVR group. Six patients (55%) in the IPVR group of the INVITE trial required CPB [21]. In our study, however, no CPB was needed for any patient undergoing IPVR.

In our study, valve displacement was observed in one patient in the IPVR group during the early postoperative hours, which we attribute to a technical error during implantation. This complication, which manifested as acute hemodynamic instability, was successfully managed by immediate reoperation and conversion to conventional surgical PVR. Such a displacement can be fatal if not addressed promptly and this experience underscores the critical importance of vigilant postoperative monitoring and the necessity of a rapid surgical response for potential valve-related complications. Similarly, Ghiselli et al. [22] reported valve dislocation in two patients in their published series of patients who underwent IPVR, attributing it to their nonutilization of external sutures. The single technical complication in our IPVR series occurred early in the institutional experience and highlights the learning curve associated with this technique. The complication led to important modifications in our surgical technique, including more rigorous attention to valve positioning, enhanced fixation methods with additional sutures when indicated, and more frequent use of intraoperative imaging to confirm optimal valve placement. The learning curve associated with injectable valve technology should be considered during implementation, and institutions considering adoption of this technology should ensure appropriate training and gradual implementation with careful monitoring of early results.

The excellent long-term outcomes observed in both groups, including 100% freedom from valve-related reintervention during a mean follow-up period of 10.5 years, compare favorably with published series of both conventional surgical and transcatheter PVR [10,23]. The similar long-term results suggest that both techniques are equally effective in addressing the underlying pathophysiology and achieving durable clinical outcomes.

The significant right ventricular reverse remodeling observed in both groups represents one of the most important therapeutic benefits of these approaches. The normalization of RVEDVI in the majority of patients (>85% in both groups) demonstrates that both approaches can effectively interrupt progressive ventricular dilatation when performed with appropriate timing. The similar remodeling degrees suggest that the primary determinant is regurgitation elimination rather than specific surgical approach.

Our findings align with the emerging literature on injectable valve technology while providing unique insights into pediatric applications. The early postoperative advantages observed in our series are consistent with the theoretical benefits of CPB avoidance, though few studies have provided direct comparative data from pediatric populations. The degree of right ventricular reverse remodeling achieved in both of our groups exceeds that reported in many adult series, likely reflecting the greater regenerative capacity of the pediatric myocardium and the importance of timely intervention before irreversible changes occur.

Our results have important implications for clinical practice and patient selection strategies. Injectable valve technology should be considered for patients meeting specific anatomical criteria, particularly those with annulus diameters between 15 and 30 mm and an absence of complex concomitant abnormalities. The early postoperative recovery profile associated with this technique may make this approach a viable option for reducing short-term morbidity while providing long-term hemodynamic outcomes comparable to conventional methods.

Although formal cost-effectiveness analysis was not performed in this study, the significant reductions in resource utilization observed with IPVR have important economic implications. Shorter ICU stays, reduced mechanical ventilation requirements, and decreased length of hospital stay translate to substantial cost savings, particularly in the current healthcare environment where resource optimization is increasingly important.

This study has several important limitations that must be acknowledged. The retrospective design and relatively small sample size limit the statistical power and generalizability. The nonrandomized patient selection may have introduced selection bias, particularly given that CPVR patients more frequently had additional cardiac abnormalities requiring surgical correction. The heterogeneous nature of the CPVR group, with some patients undergoing additional procedures, may also have influenced early outcome comparisons. However, the specific patient selection criteria required for the feasibility of the injectable technique, as detailed in Section 2, precluded a homogeneous distribution between the study groups. Consequently, regarding operative and early postoperative outcomes, this study should be interpreted as a report of our institutional experience with both conventional and novel techniques rather than a direct comparative analysis of two equivalent groups. A comparative evaluation remains more appropriate for long-term valve performance and durability. Our results demonstrate that injectable valves achieve success comparable to that of conventional prostheses in the mid-to-long term; however, further studies with larger patient cohorts are necessary to validate these findings. The absence of cardiac magnetic resonance imaging for objective assessment represents another limitation of our study. Future studies should address these limitations with prospective randomized trial designs, larger patient populations, and more comprehensive imaging assessment.

## 5. Conclusion

Injectable bioprosthetic PVR appears to be a safe and feasible alternative for treating PR following TOF repair. While it offers favorable early postoperative recovery, its long-term hemodynamic performance, prosthesis durability, and impact on right ventricular remodeling remain comparable to those of the conventional technique. However, careful patient selection based on anatomical criteria, adequate surgical experience, and attention to technical details are essential for optimal outcomes. The choice between injectable and conventional techniques should be individualized based on anatomical considerations, patient factors, and institutional experience.

## Figures and Tables

**Table 1:** Patient demographics and baseline characteristics.

Variable	IPVR (n=9)	CPVR (n=13)	p-value
Age (years)	10.3 ± 3.2	11.2 ± 2.4	0.503
Male sex, n (%)	6 (66.7)	7 (53.8)	0.695
Weight (kg)	35.2 ± 12.8	38.9 ± 11.2	0.456
BSA (m^2^)	1.18 ± 0.28	1.26 ± 0.24	0.442
Symptomatic presentation, n (%)	7 (77.8)	11 (84.6)	1.000
Exercise intolerance, n (%)	6 (66.7)	9 (69.2)	1.000
Dyspnea, n (%)	4 (44.4)	8 (61.5)	0.451
Preoperative RVEDVI (mL/m^2^)	142.3 ± 30.2	157.5 ± 23.0	0.195
Preoperative RVESVI (mL/m^2^)	90.0 ± 16.6	111.1 ± 20.6	0.019*
Pulmonary annulus diameter (mm)	24.8 ± 3.2	22.1 ± 2.8	0.045*
Time to reoperation† (years), median (IQR)	8.0 (2.0)	9.0 (5.0)	0.591

BSA: Body surface area; RVEDVI: right ventricular end-diastolic volume index; RVESVI: right ventricular end-systolic volume index;

† time between initial operation and pulmonary valve replacement;

* statistically significant.

**Table 2:** Operative characteristics.

Variable	IPVR (n=9)	CPVR (n=13)	p-value
CPB time (min)	0	77.0 ± 17.4	<0.001*
Cross-clamp time (min)	0	52.3 ± 14.2	<0.001*
Implanted valve size (mm)	26.1 ± 3.8	23.6 ± 2.8	0.089
Concomitant procedures, n (%)	0 (0.0)	10 (76.9)	<0.001*
- VSD closure	0	3	-
- PA stenosis repair	0	5	-
- Tricuspid annuloplasty	0	2	-

CPB: Cardiopulmonary bypass; VSD: ventricular septal defect; PA: pulmonary artery.

**Table 3:** Early postoperative outcomes.

Variable	IPVR (n=9)	CPVR (n=13)	p-value
ICU stay (h)	16.8 ± 6.2	37.0 ± 23.4	0.021*
Mechanical ventilation (h)	5.2 ± 3.9	15.4 ± 11.4	0.019*
Extubation within 6 h, n (%)	8 (88.9)	5 (38.5)	0.020*
Chest tube drainage (mL)	206.7 ± 108.2	513.1 ± 274.1	0.005*
Hospital stay (days)	5.4 ± 2.4	8.4 ± 3.1	0.026*
Blood transfusion, n (%)	1 (11.1)	8 (61.5)	0.020*
Inotropic support score	2.1 ± 1.8	4.8 ± 3.2	0.032*
Complications, n (%)	0 (0.0)	5 (38.5)	0.043*
- Prolonged ventilation	0	2	-
- Renal dysfunction	0	1	-
- Wound infection	0	1	-
- Heart block	0	1	-
Mortality, n (%)	0 (0.0)	0 (0.0)	1.000

ICU: Intensive care unit.

**Table 4:** Long-term outcomes.

Variable	IPVR (n=9)	CPVR (n=13)	p-value
Follow-up duration (years)	10.3 ± 3.0	10.7 ± 2.3	0.751
Postoperative RVEDVI (mL/m^2^)	94.1 ± 17.8	103.2 ± 19.9	0.288
Postoperative RVESVI (mL/m^2^)	59.1 ± 12.0	67.8 ± 13.1	0.127
RVEDVI reduction (mL/m^2^)	48.2 ± 25.1	54.3 ± 18.9	0.521
RVEDVI <120 mL/m^2^, n (%)	8 (88.9)	10 (76.9)	0.628
Ejection fraction (%)	65.1 ± 3.7	61.3 ± 5.5	0.087
Transvalvular gradient (mmHg)	25.6 ± 9.2	30.9 ± 16.2	0.381
Pulmonary regurgitation grade			
- None/trivial, n (%)	7 (77.8)	9 (69.2)	0.695
- Mild, n (%)	2 (22.2)	4 (30.8)	0.695
- Moderate/severe, n (%)	0 (0.0)	0 (0.0)	1.000
NYHA Class I, n (%)	8 (88.9)	11 (84.6)	1.000
Arrhythmias, n (%)	0 (0.0)	0 (0.0)	1.000
Reintervention, n (%)	0 (0.0)	0 (0.0)	1.000

RVEDVI: Right ventricular end-diastolic volume index; RVESVI: right ventricular end-systolic volume index.
